# Effect of an Intervention Program on Gross Motor Skills in Peruvian Preschool Children

**DOI:** 10.3390/children13091210

**Published:** 2026-09-08

**Authors:** Joel Figueroa-Quiñones, Julissa Pariona Espino, Ashley Oliveros Marquez, Cesar Ortiz Huamán, Kerrily Fiorell Fernández Castilla, Dayanna Nayelly Pérez Calderón, Juan Carlos Bazo-Alvarez

**Affiliations:** 1 School of Psychology, Faculty of Health Sciences, Universidad Autónoma de Ica, Chincha Alta 11701, Peru; 2 Research Department of Primary Care and Population Health, University College London, London NW3 2PF, UK

**Keywords:** gross motor skills, preschool children, intervention program, TGMD-3, early childhood development

## Abstract

**Highlights:**

**What are the main findings?**

• An eight-session motor circuit-based intervention produced significant improvements in overall and locomotor motor skills in Peruvian five-year-old children.
• The experimental group improved by more than 10 points in overall motor skills compared to the control group.

**What are the implications of the main findings?**

• Short, structured motor intervention programs can effectively enhance fundamental motor skills during early childhood.
• These findings support the inclusion of motor circuit-based activities in preschool settings to promote physical development and long-term well-being.

**Abstract:**

**Background:** The development of motor skills in childhood contributes to overall well-being throughout life. However, delays in these skills without intervention may negatively affect both physical and psychological health. Objective: This study aimed to evaluate the effect of an intervention program on gross motor skills in Peruvian preschool children. **Methods:** A quasi-experimental study was conducted with a sample of 45 five-year-old children assigned to a control group (n = 24) and an experimental group (n = 21). The intervention consisted of an eight-session program of 45 min physical activities delivered over six weeks. The TGMD-3 was administered before and after the intervention in both groups. The data were analyzed through comparisons of pre–post group differences with *p* < 0.05. In addition, a repeated-measures mixed-model analysis was conducted, reporting β coefficients, standard errors, and 95% confidence intervals, with adjustment for confounding variables. **Results:** The experimental group showed a significant average increase of 10.67 points in overall motor skills and 6.67 points in locomotor skills compared to the control group. Moreover, the mixed models confirmed significant Time × Group interactions for both overall motor skills (β = 10.8, SE = 4.6, 95% CI [1.7, 19.8]) and locomotor skills (β = 6.2, SE = 2.3, 95% CI [1.7, 10.7]). **Conclusions:** The intervention program significantly improved fundamental motor skills in five-year-old children. In this regard, motor circuit-based programs represent an effective strategy to support early childhood development.

## 1. Introduction

Fundamental motor skills are essential components of human life, as they enable the performance of everyday and sports activities [1]. These skills begin to develop from the very first days of life through small movements, such as seeking the mother’s breast for feeding, moving the fingers and toes, or holding the head and trunk while sitting [2]. In the following years, individuals acquire the ability to perform more complex movements such as jumping, running, catching, or kicking a ball, which are known as gross motor skills [3]. Several studies have demonstrated a close relationship between motor development, overall well-being, and quality of life [4].

A global report indicates that most children today exhibit below-average levels of motor development compared to normative data collected during the 1990s [5]. In this regard, children worldwide are showing limitations in their motor development, which may be detrimental to both their physical and mental health. For example, a study conducted with children in Germany found that lower levels of gross motor development were associated with obesity and overweight problems [6]. Similarly, a study of Danish children revealed that those with poorer fine and gross motor skills reported lower performance in mathematical ability and reading comprehension [7]. Moreover, a study with English children reported that those with developmental difficulties had a higher likelihood of developing depression [8].

In several countries, various strategies have been implemented to promote motor development and improve children’s health [9]. For example, in the United States, a study implementing a motor activity program found evidence of improvements in children’s health [10]. Similarly, in Australia, a study reported medium effect sizes in the experimental group, with higher scores in gross motor skills [11]. In Hong Kong, another study concluded that implementing a school-based fundamental movement skills training program can promote both physical and psychological health in children [12]. In Latin America, however, a review revealed that interventions aimed at improving children’s motor skills often lack a rigorous evaluation and intervention process, highlighting the need for stronger evidence regarding the effectiveness of physical education programs in educational institutions [13].

To promote motor development in early childhood, motor circuits based on functional training represent an effective alternative [14]. Functional training adopts an approach centered on movements such as pushing, pulling, jumping, turning, and moving through space, with the purpose of improving the body’s efficiency in daily activities [15]. Unlike isolated or repetitive exercises, this approach integrates multiple muscle groups and simultaneously stimulates strength, balance, coordination, and stability, which are essential components for the development of gross motor skills in young children [16]. Likewise, these activities are organized in the form of circuits, creating brief and varied stations that are playful, challenging, and easy to adjust progressively [17]. In fact, recent research has reported that programs based on functional training lead to significant improvements in locomotor and object-control skills, as well as increased active participation and motivation among children [14,18]. Moreover, its flexible design allows implementation in schools with limited space or minimal equipment, positioning this approach as a viable alternative for interventions aimed at strengthening motor performance in preschool children [19].

In Peru, several contextual conditions may hinder the normal development of basic motor skills in preschool-aged children. For example, it has been established that the availability of facilities and adequate physical space plays a key role in motor development [20]. However, most public schools in the country face a lack of appropriate infrastructure [21]. In addition, national health data indicate that 8.6% of children under five years old and three out of ten adolescents are overweight or obese [22,23], which may further restrict children’s physical activity and motor progress. A Peruvian study on children aged 6 to 10 years found below-average motor competence levels (17.5%), particularly among girls [24]. Likewise, Figueroa-Quiñones et al. [25] found that younger girls living in urban environments exhibited poorer motor performance, emphasizing the need for targeted and contextually adapted interventions. Despite these findings, no previous study in Peru has implemented or evaluated a structured intervention program specifically aimed at enhancing motor development in preschool children.

Therefore, our study aimed to evaluate the effect of an intervention program on gross motor skills in Peruvian preschool children.

## 2. Materials and Methods

### 2.1. Design

The study used a quasi-experimental design with repeated measures (pre-test and post-test), including an experimental group (EG) and a control group (CG). The EG received an intervention program, while the CG followed their regular physical education curriculum.

### 2.2. Participants

**Figure 1:**
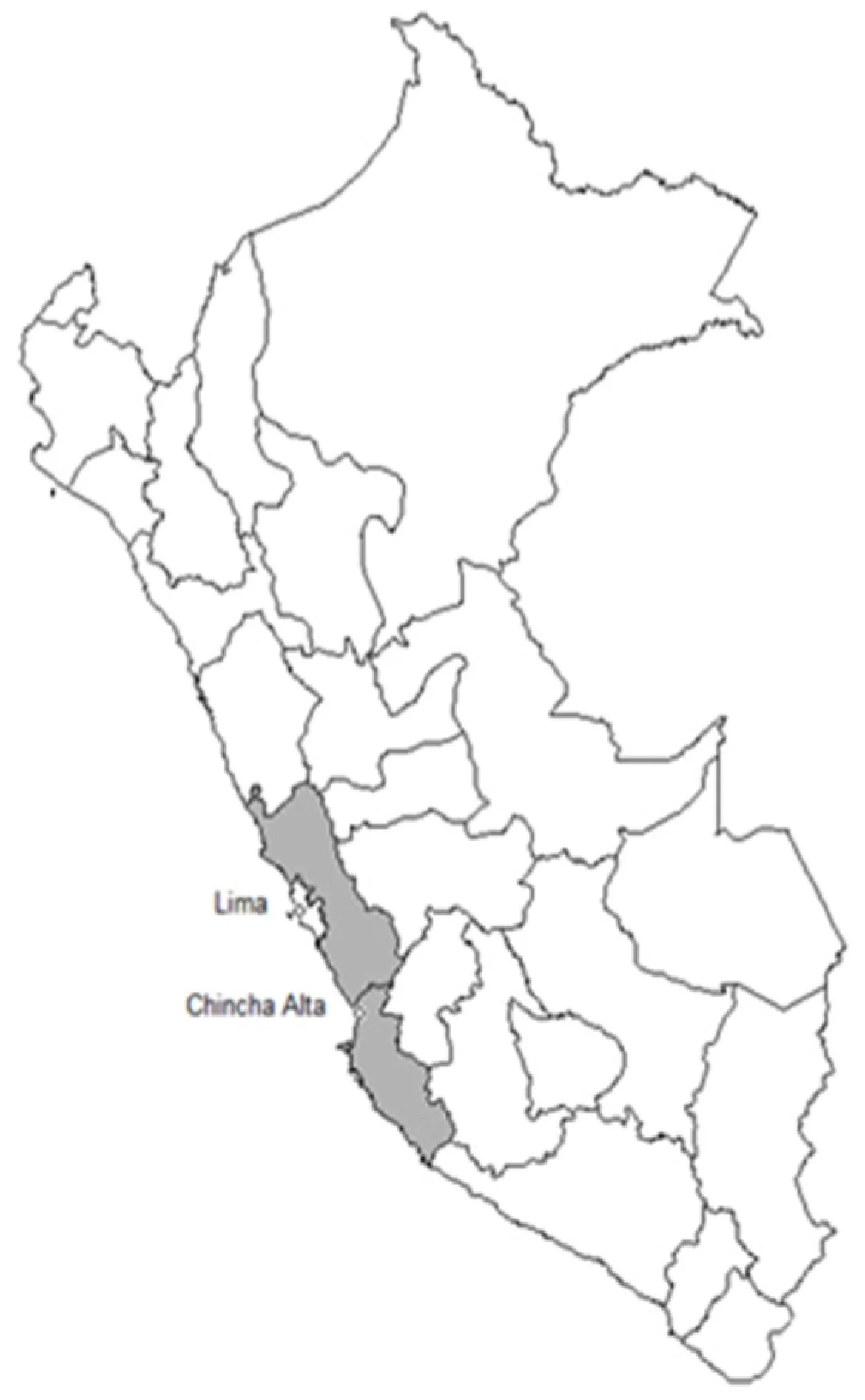
Location of Chincha Alta in the Ica region, Peru.

The study was conducted in the city of Chincha Alta, located in the Ica region, approximately 200 km south of Lima, the capital of Peru (see Figure 1). Chincha Alta has a warm climate and an altitude of 95.0 m above sea level. In addition, it has a population of more than 82,000 inhabitants and an economy based on agricultural exports, commerce, and tourism due to its Afro-Peruvian cultural heritage [26].

**Figure 2:**
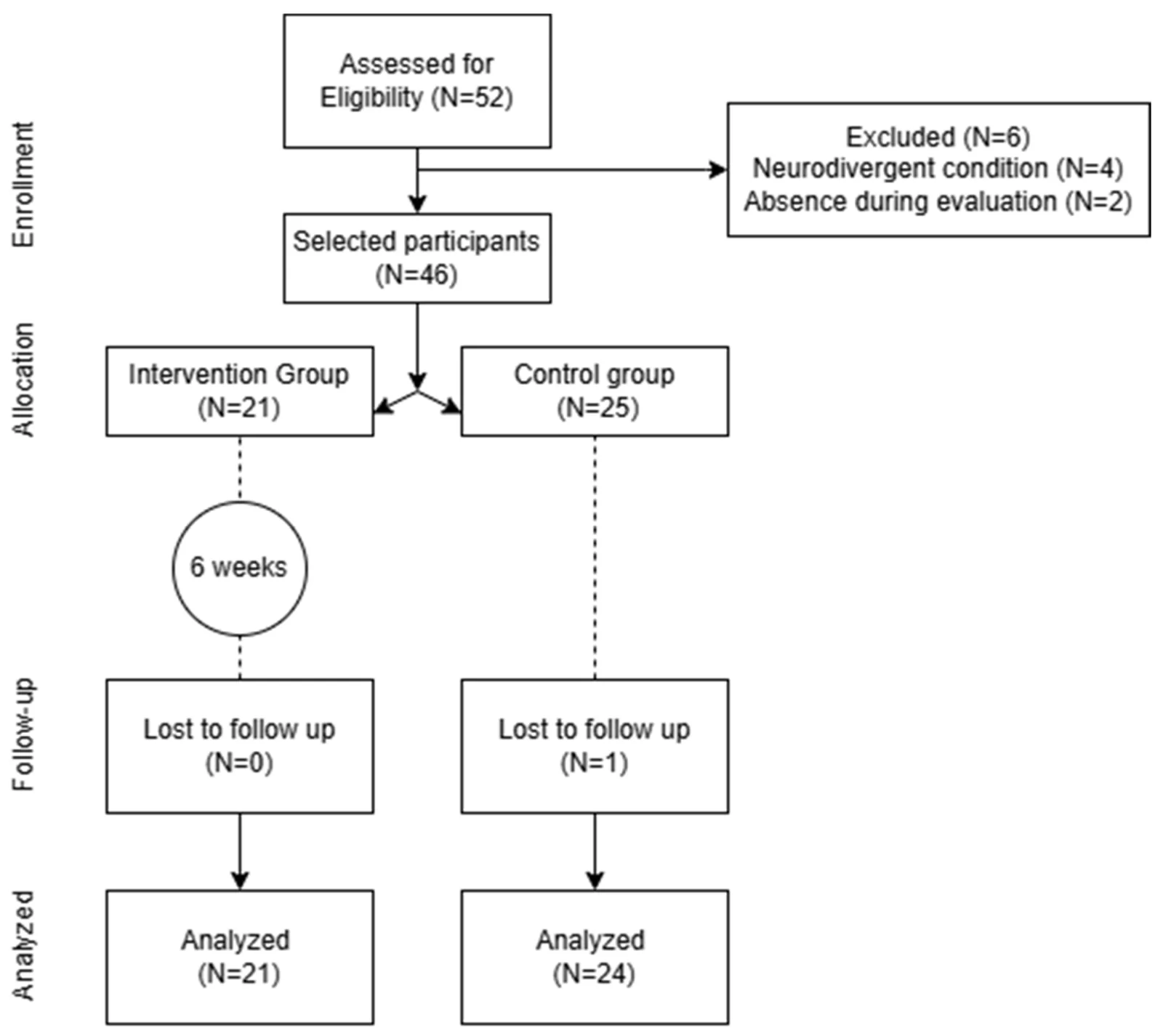
CONSORT flow diagram.

A total of 52 participants were recruited, of whom 46 preschoolers (26 boys and 20 girls) aged 5 years were included in the study (CG: Mage months = 67.7, SD = 3.49; EG: Mage months = 68, SD = 3.42). Participants were drawn from a public early childhood education institution. Participants were assigned based on pre-existing classrooms, without individual randomization. Both classrooms belonged to the same institution and corresponded to the five-year-old level of early childhood education. Each classroom was led by a different teacher but followed the same national curriculum under comparable educational conditions. Inclusion criteria required parental permission through informed consent and the child’s subsequent assent. Children presenting any neurodivergent condition or physical limitation, as recorded in school enrollment documents or reported by the teacher, were excluded. Likewise, those absent during the evaluation phase were not included. Figure 2 illustrates the study flow diagram, and Table 1 presents the demographic characteristics of the participants

### 2.3. Intervention

The activities of the program for developing fundamental gross motor skills in preschoolers were adapted from the functional training program proposed by Dong Zhang et al. [19]. Accordingly, the intervention program was culturally adapted and contextualized for Peruvian preschool children using the ADAPT-ITT model [27]. This model follows eight guided phases for systematic adaptation. An expert review was conducted (including one public-sector child sports specialist, one private-school physical education teacher, one early childhood education teacher, and one clinical psychologist with experience in child motor stimulation), along with pilot testing to refine the content and ensure its feasibility within the local educational context. In addition, teachers and research assistants received training at the school from a child sports specialist on the implementation of the program, and the accuracy of the intervention protocol was evaluated. A detailed description of the adaptation process is provided in Appendix A.

The final adapted program was structured into three phases per session: (a) a five-minute warm-up with stretching activities aimed at preparing the body and fostering a readiness for movement; (b) a thirty-five-minute main activity phase, consisting of physical exercises designed to strengthen fundamental motor skills; and (c) a five-minute cool-down with stretching movements to promote recovery. In total, the program was designed as an eight-session intervention applied over six weeks, with each session lasting 45 min. Supplementary Material S1 provides a detailed description of the intervention structure.

The program was implemented by the children’s lead teacher and conducted twice a week. The teacher was supported by assistants, who helped with the setup and removal of equipment, and a research leader, who supervised the timing and application of each activity and recorded participant attendance during the sessions in the preschool’s psychomotor room.

### 2.4. Procedure

The study was carried out in four phases: (1) the institutional identification phase, (2) the pretest assessment, (3) the intervention period, and (4) the posttest assessment (see Figure 2).

In the first phase, the director of the Local Educational Management Unit (UGEL) of the city was contacted through the official correspondence system. The UGEL identified a public preschool institution that met the necessary criteria and invited it to participate in the study. The institution was selected based on its preschool level, the availability of a large open area suitable for physical activities without interruptions, and a secure space for storing materials and equipment throughout the study period.

Subsequently, the research team met exclusively with the teachers of the selected classrooms to explain the intervention procedures in detail. The teachers assisted in distributing informed consent forms to parents and/or guardians through the institution’s WhatsApp communication groups. Verbal assent was then obtained from each child prior to participation.

In the second phase, the Test of Gross Motor Development—Third Edition (TGMD-3) was administered. Each assessment was video-recorded using a Canon R50 camera (Canon Inc., Tokyo, Japan) with an RF 18–45 mm lens, positioned frontally at a distance of 4.5 m, with adequate lighting and mounted at a height of 1.10 m on a Weifeng WF-3950 tripod (Weifeng Group, Zhongshan, China).

Children were evaluated individually in the schoolyard during the afternoon shift, accompanied by the classroom assistant. The assessments were conducted by the study leader and four trained research assistants, senior psychology students who had received prior training in data collection procedures. All assessments were carried out over a six-week period between May and July 2025, under standardized conditions. Only children whose parents had provided informed consent and who assented to participate were assessed.

The third phase consisted of implementing the intervention program. This stage was carried out over six weeks in a motor skills classroom within the preschool’s facilities. The sessions were conducted on Tuesday and Thursday afternoons, according to the schedule previously agreed upon with the preschool teachers and coordinators. The EG participated in the intervention sessions led by the classroom teacher, while the CG continued with their regular physical education classes. Prior to the intervention, the classroom teacher received structured training on the program procedures and was provided with a written guide detailing each session. During implementation, research assistants supported attendance control and material setup, following the structured guide designed to standardize session delivery. The principal investigator was present during all sessions to supervise adherence to the protocol and provide assistance when required. A predefined schedule was maintained to document session implementation, and the teacher was instructed to follow the program as designed without modifying the activities.

In the fourth phase, the same procedures and instruments used in the pre-test were applied to ensure consistency and comparability of the data. Accordingly, during this stage, the TGMD-3 was administered to each child individually in the afternoon, with the participation of the project leader and four trained research assistants, in the presence of the classroom assistant. All assessments were completed within three weeks following the conclusion of the intervention program.

### 2.5. Measures

The following anthropometric measurements were taken using equipment certified by the Ministry of Health of Peru (MINSA): body weight and body mass index (BMI) were measured with an OMRON HBF-514C body composition scale (Omron Healthcare Co., Ltd., Kyoto, Japan), and height was measured with a portable wooden stadiometer. In addition, several covariates were included, such as age (in months), sex (male and female), and maternal education (primary, secondary, and university level).

The Test of Gross Motor Development–Third Edition (TGMD-3) was used. This version of the test was developed by Ulrich et al. in the United States in 2018 and assesses fundamental motor skills based on the quality of performance in six locomotor skills (running, hopping, sliding, galloping, single-leg jump, and two-leg jump) and six object control skills (kicking a ball, dribbling, overhand and underhand throwing, catching a ball with the hands, and striking a ball with a bat). The administration time ranges from 10 to 15 min. Each skill is scored with 1 point if the child performs the criterion correctly in either of the two trials; otherwise, it receives 0 points. The TGMD-3 has demonstrated intraclass correlation coefficients above 0.88 for preschool-aged children and Pearson correlations greater than 0.91 when compared with similar motor development tests.

In Peru, the TGMD-3 has shown adequate psychometric properties in children, with internal consistency (α = 0.851), inter-rater reliability (0.963), and intra-rater reliability (0.983) [24]. Figueroa et al. [25] reported optimal fit indices in the two-factor model, with α values > 0.8 and invariance between groups of children aged 3 to 6 years. Furthermore, recent studies in Peru have applied this version to assess preschool motor performance.

### 2.6. Data Analysis

A descriptive statistical analysis was conducted for the sociodemographic variables and fundamental motor skill problems, which were presented in frequency tables and as means with their corresponding standard deviations. Baseline equivalence between groups was examined using independent-samples *t*-tests for continuous variables and chi-square tests for categorical variables. Additionally, independent pre–post change analyses were performed for each group (control and experimental), obtaining the mean differences between baseline and post-intervention scores. For each variable, 95% confidence intervals were calculated. Between-group comparisons of pre–post differences were carried out using significance tests (*p*-values), following statistical significance criteria when *p* < 0.05. Finally, to assess the effect of the program over time and group membership, a mixed-model repeated-measures analysis was employed to examine the Time × Group interaction for overall motor skills and their subscales. The model included β coefficients, standard errors, and 95% confidence intervals, and was adjusted for sex, BMI, and age in months. All data were analyzed using StataCorp v17.0 and RStudio version 2024.09.1+394.

## 3. Results

Table 1 presents the baseline characteristics of the participants (n = 46). Following the non-random allocation of children to the control group (CG) and the experimental group (EG), the mean age was comparable between groups. The most frequent age range for the CG was 62–64 months, whereas for the EG it was 68–70 months. On average, children in the EG showed slightly higher weight and height compared to those in the CG, while the mean BMI was identical in both groups. The sex distribution indicated a higher proportion of boys in the CG, whereas girls were slightly more represented in the EG. In both groups, most mothers (>60%) had completed secondary education. Regarding motor skills, the majority of children in both groups demonstrated a very low level of overall motor performance (>70%), locomotor skills (>71%), and ball skills (>67%). No statistically significant differences were observed between groups at baseline (all *p* > 0.05)

Table 2 shows that the experimental group (EG) exhibited an average increase in overall motor skills of 10.6 points (SD = 13.7; 95% CI [4.4, 16.9]), whereas the control group (CG) remained unchanged (M = −0.0; SD = 16.9; 95% CI [−7.2, 7.0]). The difference in mean changes between the two groups was statistically significant (*p* = 0.025).

Similarly, locomotor skills improved by 6.6 points in the EG (SD = 6.8; 95% CI [3.5, 9.7]) compared to 0.4 points in the CG (SD = 8.3; 95% CI [−3.0, 3.9]), also showing a significant between-group difference (*p* = 0.010).

Regarding ball skills, although the EG showed an average increase of 4.0 points and the CG a slight decrease of −0.5, the between-group differences did not reach statistical significance (*p* = 0.106).

**Figure 3:**
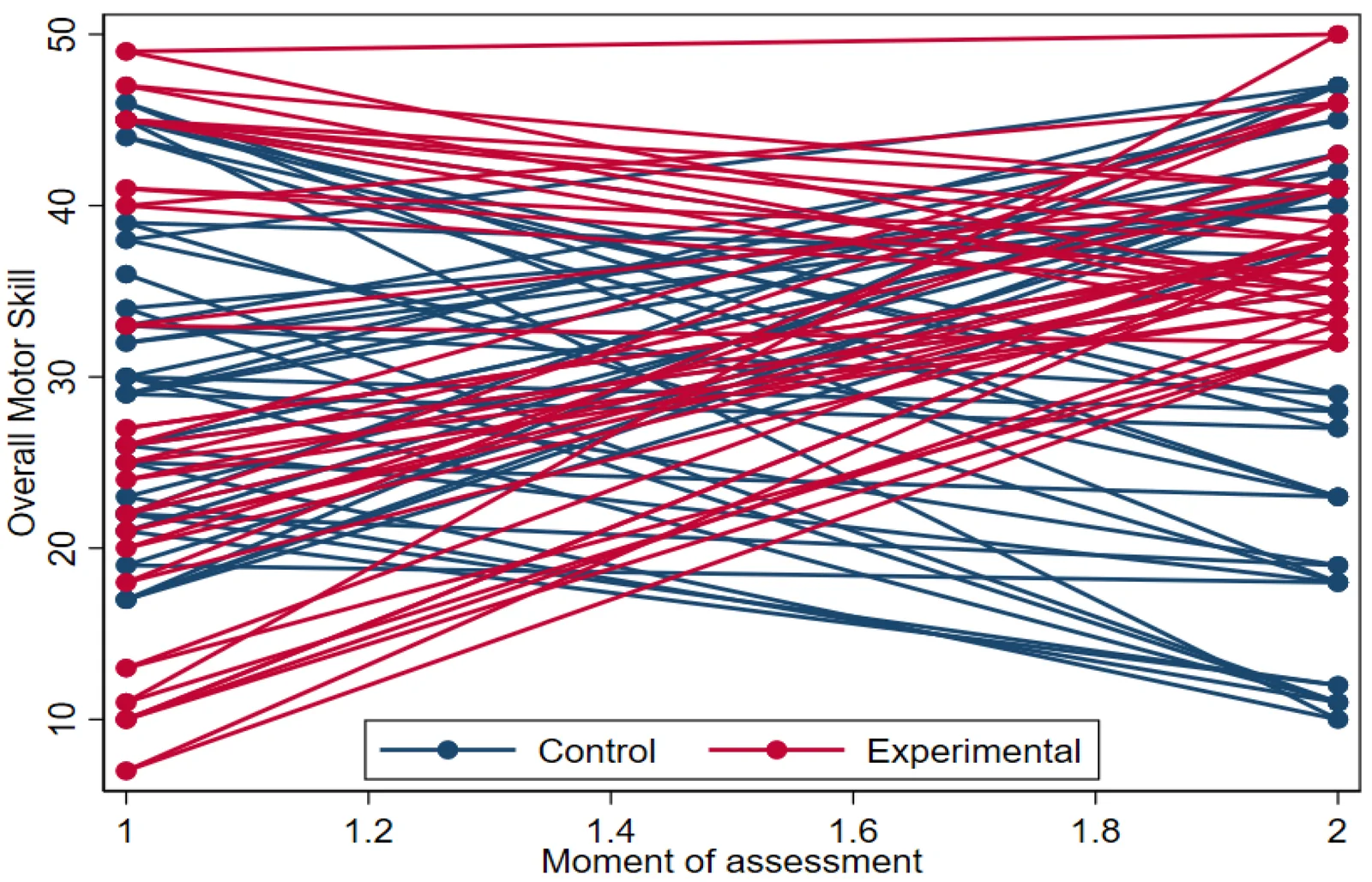
Spaghetti Plot: Individual Trajectories of Overall Motor Skill by Group.

**Figure 4:**
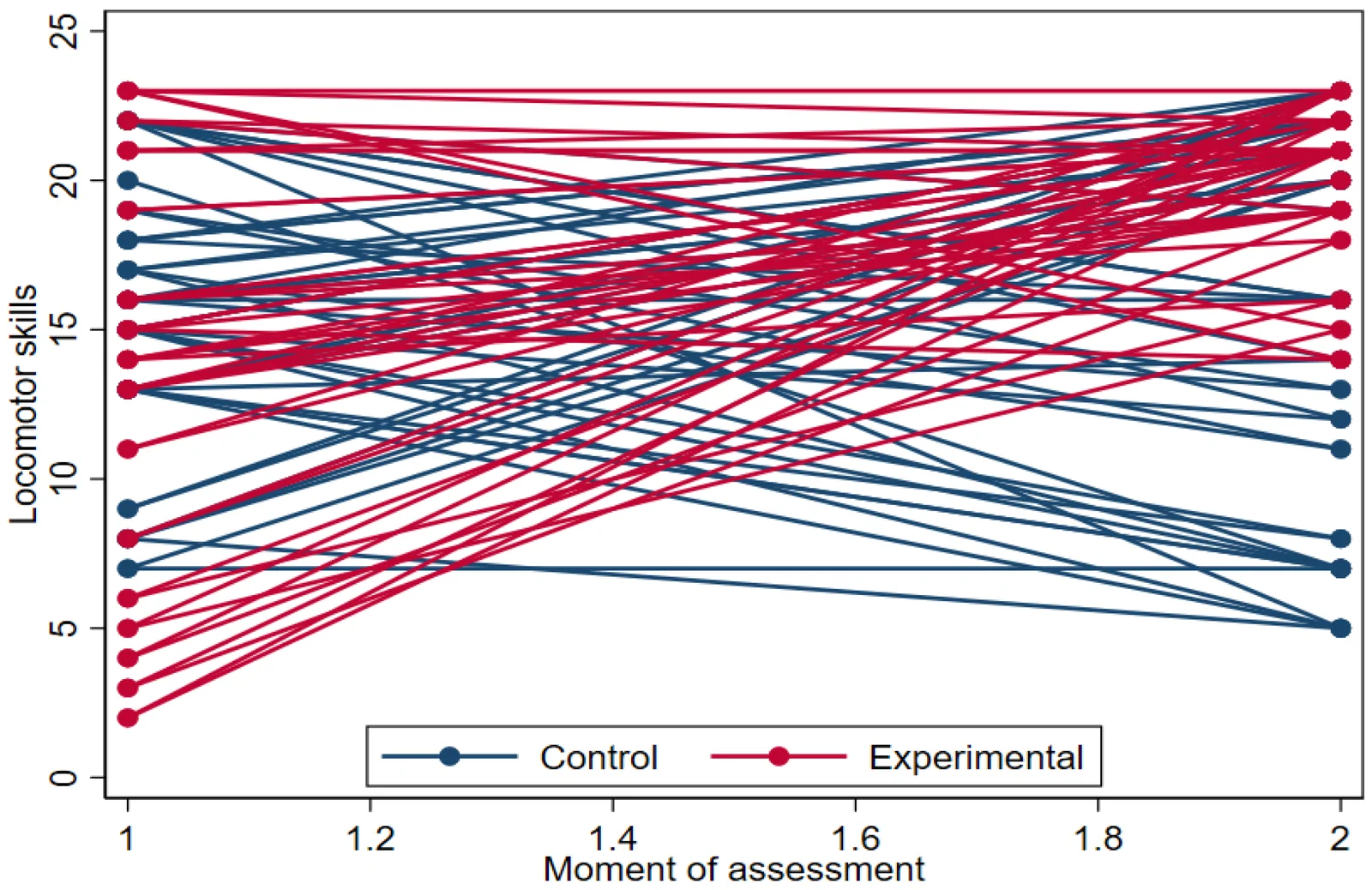
Spaghetti Plot: Individual Trajectories of Locomotor by Group.

**Figure 5:**
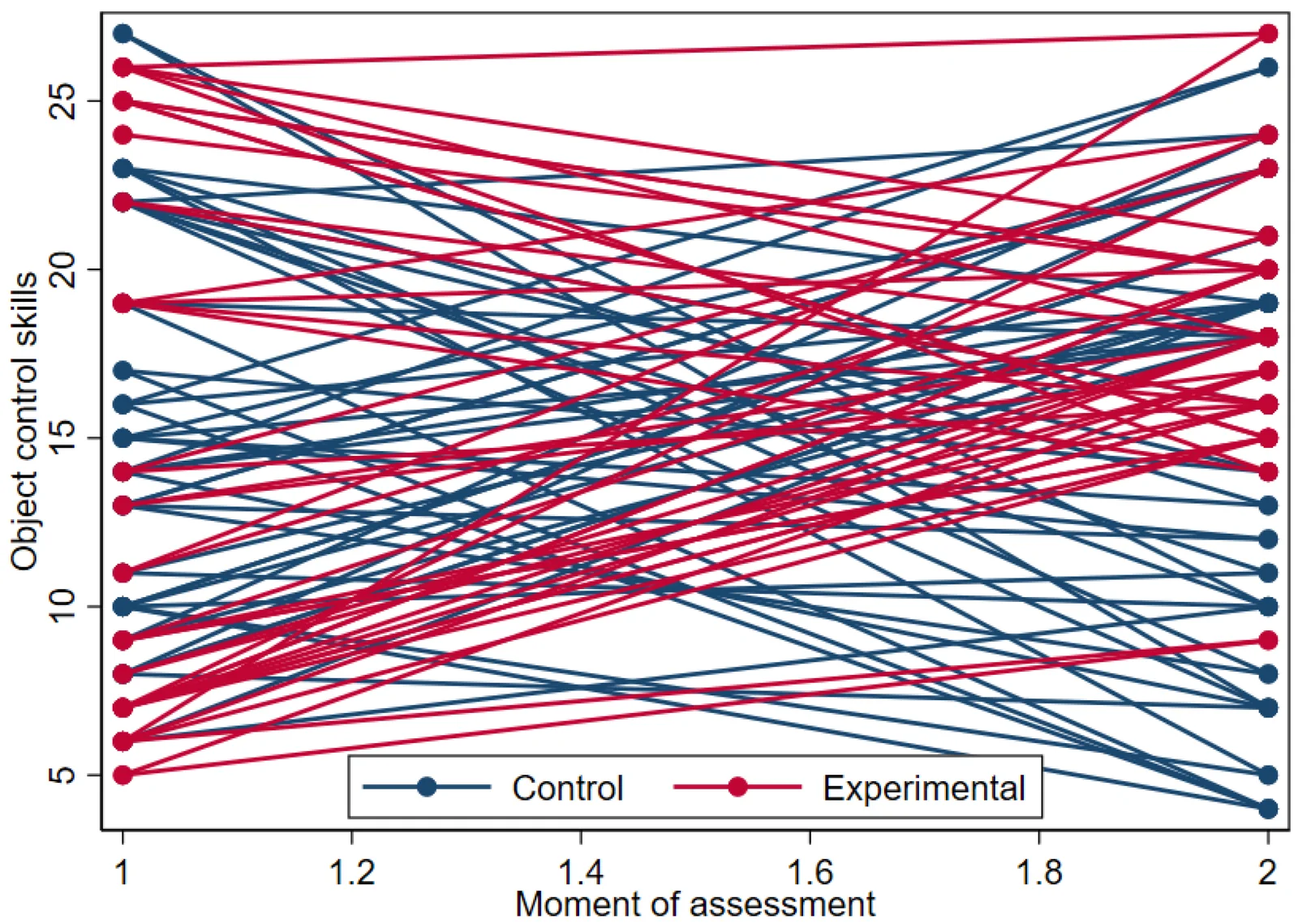
Spaghetti Plot: Individual Trajectories of Ball Skills by Group.

Figure 3, Figure 4 and Figure 5 present spaghetti plots for each motor skill domain, showing more consistent upward individual changes in the EG compared to the CG.

As shown in Table 3, significant Time × Group interaction was observed for both overall motor skills (β = 10.8, SE = 4.6, 95% CI [1.7, 19.8], *p* = 0.020) and locomotor skills (β = 6.2, SE = 2.3, 95% CI [1.7, 10.7], *p* = 0.006), indicating that the EG showed significantly greater improvement over time compared to the CG. In contrast, no statistically significant interaction was found for ball skills (β = 4.5, SE = 2.7, 95% CI [−0.7, 9.8], *p* = 0.090), indicating insufficient evidence to conclude a differential effect between groups. All analyses were adjusted for sex, body mass index (BMI), and age (in months).

## 4. Discussion

This quasi-experimental study implemented a six-week physical activity-based intervention aimed at enhancing motor skill development in Peruvian preschool children. After the intervention, the experimental group (EG) showed significantly greater improvements in overall and locomotor motor skills compared to the control group (CG). These effects remained significant after adjusting for sex, age, and body mass index (BMI). The program was delivered by the classroom teacher twice a week as part of the regular school schedule. These findings support existing evidence that structured physical activity programs can effectively enhance children’s motor skills.

The main finding of the study was a significant improvement in overall motor skills among children in the intervention group. Specifically, the program enhanced various locomotor and object control abilities, such as running, jumping, and kicking a ball, among other fundamental motor activities. These findings are consistent with previous research. For instance, a recent study by Dong Zhang et al. [19] involving Chinese children aged 6 to 7 demonstrated that, following a twelve-week functional training program (40 min per session), participants showed significant gains in fundamental motor skills. Likewise, a study with American children aged 5 to 8 reported that a twelve-week physical activity program led to notable improvements in general motor performance [28]. Furthermore, a recent systematic review confirmed that intervention programs based on aerobic physical activity are effective in enhancing motor development during childhood [29]. Although our program was shorter in duration (six weeks), it was implemented twice per week for 45 min and consisted of motor circuits including aerobic activities such as running and zigzag jogging between cones, jumping through hoops, and leaping over bars and hurdles of different heights. Such exercises are aligned with theoretical frameworks that emphasize the stimulation of gross motor development through repeated practice, dynamic coordination, and postural control, reinforcing the importance of structured motor interventions in early childhood.

Our program demonstrated a significant improvement in locomotor skills among children in the experimental group. This finding is consistent with previous evidence supporting the effectiveness of physical activity-based interventions in enhancing motor development. In this regard, Pham et al. [30] implemented a physical activity program with seven-year-old Vietnamese children, conducted twice per week, and observed that participants in the experimental group achieved significantly higher scores in locomotor skills. Similarly, Kelly et al. [31] conducted an eight-week physical intervention with Irish children and found significant improvements in locomotor skills, with no interaction effects related to time, sex, or weight. Moreover, a recent review concluded that physical activity-based programs represent one of the most effective strategies for promoting meaningful improvements in these abilities [19]. These results can be better understood through the theoretical perspective emphasizing the role of systematic and varied practice in the acquisition and consolidation of motor patterns. The sessions in our program included circuits designed to stimulate coordinated movement, speed, and agility, core components of locomotor development according to the model proposed by Gallahue, Ozmun, and Goodway [32], who argue that structured and repetitive practice fosters maturation and progression toward more complex movements. Complementarily, Payne & Isaacs [33] suggest that controlled repetition of movement promotes neuromuscular coordination, spatial organization, and postural control processes essential for mastering locomotor skills. Taken together, the motor circuit-based approach used in this study facilitated progressive learning through exploration, execution, and continuous feedback, highlighting the effectiveness of brief and structured programs in educational settings.

Regarding ball skills, although the experimental group showed an average improvement, this change did not reach statistical significance. These findings differ from those reported in a study conducted with Chinese children, in which significant improvements in object-control skills were observed among children aged 3 to 6 years [34]. Similarly, another study conducted in the United States reported significantly greater improvements in object-control skills in the intervention group compared to controls. Notably, both studies implemented interventions of longer duration and higher session frequency than the six-week program applied in the present study, which may have contributed to their significant outcomes. In contrast, other intervention studies have reported minimal or non-significant improvements in object-control skills; for example, a review identified six studies in which gains in object control following intervention were limited [19]. This finding may be explained by the program’s stronger emphasis on locomotor skill development, whereas tasks targeting object control such as throwing, catching, or kicking were less frequent. From the perspective of motor learning theory, Schmidt & Lee [28] argue that the acquisition of manipulative skills requires specific, varied, and continuous practice to refine control mechanisms and sensorimotor feedback. In addition, Haywood & Getchell [29] suggest that developing these skills demands activities that promote hand-eye coordination and movement precision. From a complementary perspective, Clark [35] emphasizes that progress in mastering object control skills depends on the interaction between individual characteristics, task demands, and environmental opportunities. In this regard, the focus of our program on locomotor activities may have primarily enhanced locomotor development, resulting in a differential focus of skill development rather than a lack of impact. Therefore, future interventions should incorporate a broader range of manipulative tasks within motor circuits, encouraging active exploration and structured practice in diverse contexts to strengthen motor coordination and optimize object control.

The findings of this study demonstrate the potential of school-based motor circuit programs to strengthen fundamental motor skills during early childhood. Given the characteristics of the intervention, such programs can be implemented by classroom teachers who have received prior training, without necessarily requiring specialized instructors. Moreover, integrating these sessions into the regular school schedule aligns with the national curriculum and is feasible even in resource-limited settings, such as those in low- and middle-income countries. Incorporating structured physical activity sessions into children’s weekly routines not only promotes physical and motor development but also enhances their overall well-being and readiness for learning, as supported by previous evidence [36].

In the educational and public policy sphere, the results of this study highlight the need for the Peruvian Ministry of Education (MINEDU), in coordination with the Regional Directorates of Education (DRE) and Local Educational Management Units (UGEL), to prioritize the inclusion of structured, evidence-based programs for motor development within early childhood education strategies. Furthermore, it is recommended to strengthen teacher training through professional development programs that promote physical literacy and the design of sessions focused on motor skill development. These initiatives represent sustainable, low-cost, and high-impact interventions capable of fostering active lifestyles among both educators and preschool children.

This study represents the first intervention conducted in Peru to provide scientific evidence on a standardized motor circuit program designed to strengthen fundamental motor skills, thereby contributing to the field of early motor development. A notable strength is the inclusion of a population rarely examined in intervention studies preschool-aged children within a specific cultural, geographical, and socioeconomic context. Additionally, the implementation of the program by classroom teachers in real school settings enhances external validity and demonstrates the practical applicability of the model under everyday educational conditions. However, several limitations should be acknowledged. Participants were assigned to pre-existing classrooms without randomization, which may introduce selection bias and warrants cautious interpretation. Furthermore, although both classrooms operated under comparable structural conditions, potential differences in teaching style between teachers may have influenced the findings. The sample size may have limited the statistical power to detect small-to-moderate effects, particularly in object-control skills, and should be considered when interpreting non-significant findings. Additionally, this was a monocentric study conducted in a single public early childhood institution in an urban context (Chincha Alta), which may restrict the generalizability of the findings to settings with similar socio-contextual characteristics. Future research should include larger and more diverse samples from different regions and educational contexts. Moreover, because the control group continued only with their usual routine without an alternative comparison condition, it would be advisable for subsequent studies to incorporate different types of programs or intervention methodologies that allow for comparative evaluation and identification of the most effective strategies. Another important limitation is the absence of significant improvements in ball skills, likely due to the limited number of activities focused on object control and manipulation within the program. This suggests the need to include a broader range of tasks that promote hand-eye coordination as well as ball handling, reception, and control. Similarly, the short duration of the program may not have provided sufficient time for motor learning consolidation, potentially underestimating the true effect of the intervention; thus, future studies should evaluate longer programs and include follow-up assessments to determine sustainability. Additionally, although participants were nested within classrooms, clustering effects at the classroom level were not modeled statistically. Given that only two classrooms were included, multilevel modeling was not feasible. Future studies including a larger number of clusters should consider hierarchical analytical approaches. In addition, motor development in preschool children is influenced by multiple lifestyle and environmental factors that were not assessed in this study, including physical activity outside school, screen time, sleep patterns, nutritional habits, parental support, and access to play spaces. Future research should incorporate basic lifestyle indicators to better isolate intervention effects and strengthen causal interpretation. Finally, although supervision and a structured guide supported implementation, fidelity was not formally quantified using an adherence checklist. This limits the objective assessment of protocol compliance and suggests the need for systematic fidelity monitoring in future studies. Likewise, although the analytical models were adjusted for sex, age, and BMI, unobserved variability may persist due to uncontrolled factors such as parental support, sleep quality, or habitual physical activity levels that could influence motor development and the magnitude of the program’s effects. Accounting for these potential confounders will allow future studies to obtain more precise estimates of intervention effectiveness.

## 5. Conclusions

This quasi-experimental study demonstrated that a six-week motor circuit program implemented with Peruvian preschool children significantly improved their fundamental motor skills. Finally, the findings indicate that structured interventions implemented by teachers within the school setting represent an effective and feasible strategy to promote motor development in early childhood.

## Figures and Tables

**Table 1:** Descriptive characteristics of participants in the control and experimental groups (N = 46).

Variable		Control Group (n = 25)	Experimental Group (n = 21)
		f (%)	f (%)
Age (years) M ± SD		5.6 ± 0.3	5.7 ± 0.3
Age (months)	62–64	7 (28.0)	5 (23.8)
	65–67	5 (20.0)	4 (19.0)
	68–70	6 (24.0)	7 (33.3)
	71–73	6 (24.0)	3 (14.3)
	74–75	1 (4.0)	2 (9.5)
Anthropometric measures (M ± SD)	Size	113.1 (3.7)	113.3 (4.1)
	Weight	21.9 (2.6)	22.0 (2.3)
	BMI	17.1 (1.7)	17.1 (1.4)
Sex	Boys	16 (64.0)	10(47.6)
	Girls	9 (36.0)	11 (52.4)
Maternal Education	Primary	0 (0.0)	1 (4.8)
	Second	15 (60.0)	16 (76.2)
	University	10 (40.0)	4 (19.0)
Locomotor skills (before intervention)	Very Low	19 (76.0)	15 (71.0)
	Low	5 (20.0)	6 (29.0)
	Average	1 (4.0)	0 (0.0)
Ball skills (before intervention)	Very Low	19 (76.0)	14 (67.0)
	Low	5 (20.0)	7 (33.0)
	Average	1 (4.0)	0 (0.0)
Overall Motor Skill (before intervention)	Very Low	19 (76.0)	15 (71.0)
	Low	6 (24.0)	6 (29.0)

BMI: Body Mass Index; f: frequency; M: Mean; SD: Standard Deviation; %: Percentages.

**Table 2:** Pre–post difference in the TGMD-3 between the control group (n = 24) and the experimental group (n = 21).

Variable	Group	Pre M (SD)	Post M (SD)	Difference M (SD)	95% CI	*p*-Value
Overall Motor Skill	Control (n = 24)	30.3 (9.05)	30.2 (12.65)	−0.0 (16.97)	[−7.2, 7.0]	0.025
	Experimental (n = 21)	27.5 (13.93)	38.2 (4.83)	10.6 (13.71)	[4.4, 16.9]	
Locomotor	Control (n = 24)	15.2 (4.34)	15.6 (6.42)	0.4 (8.32)	[−3.0, 3.9]	0.010
	Experimental (n = 21)	13.5 (6.74)	20.1 (2.61)	6.6 (6.84)	[3.5, 9.7]	
Ball skills	Control (n = 24)	15.1 (5.73)	14.5 (6.73)	−0.5 (9.69)	[−4.6, 3.5]	0.106
	Experimental (n = 21)	14.0 (7.88)	18.0 (3.90)	4.0 (8.61)	[−0.08, 7.9]	

Note: Values represent the mean ± SD of post–pre differences within each group. 95% CI: confidence interval of the mean difference. *p*-value: compares post–pre mean differences between groups.

**Table 3:** Mixed-Model Repeated Measures Analysis of the Time × Group Interaction for Gross Motor Skills Outcomes.

Dependent Variable	Time × Group Effect (β)	SE	95% CI	*p*
Overall Motor Skill	10.75	4.61	[1.7, 19.8]	0.020
Locomotor skills	6.20	2.28	[1.7, 10.7]	0.006
Ball skills	4.54	2.68	[−0.7, 9.8]	0.090

The mixed-effects model was adjusted for sex, body mass index (BMI), and age in months.

## Data Availability

The data is available upon request to the corresponding author.

## Supplementary Materials

The following supporting information can be downloaded at: https://www.mdpi.com/article/10.3390/children13091210/s1, Supplementary Material S1. Detailed description of the intervention.

## Abbreviations

The following abbreviations are used in this manuscript:

TGMD	Test of Gross Motor Development
UGEL	Local Educational Management Unit (UGEL)
MINSA	Ministry of Health of Peru

## Appendix A. Adaptation of the Program Using the ADAPT-ITT Model

To ensure the effectiveness and cultural relevance of the program for Peruvian five-year-old children, the ADAPT-ITT model was followed, which allows evidence-based interventions to be adapted to specific populations. This process was carried out in eight phases:

(1) Evaluation:

Previous studies on the motor development status in preschool populations and improvement interventions were reviewed. The need to apply a functional training program adapted for Peruvian children in early childhood education to develop their motor skills was identified.

(2) Decision:

The functional program by Dong Zhang et al. (2024) [19] was selected as the basis due to its demonstrated effectiveness in developing fundamental motor skills in children. However, the need for adjustments was identified to align it with the Peruvian educational context.

(3) Administration:

Adjustments were made to the structure and content of the program, ensuring that the activities were culturally relevant and aligned with the Peruvian context. Pilot sessions were conducted at a public early childhood education school to assess the understanding and acceptance of the program by children and teachers. Observations and feedback identified aspects that required modification.

(4) Preparation:

Based on the feedback obtained from the previous phase, adjustments were made to the structure and content of the program to ensure that the activities were culturally relevant and aligned with the Peruvian context. The program was reviewed by experts in sports, child development, and early childhood education, who evaluated its relevance, clarity, and feasibility.

(5) Training:

In this phase, teachers and research assistants were trained in the execution of the adapted program. The training consisted of presenting the objectives and development of functional training, emphasizing the program’s direction to strengthen gross motor skills in five-year-old children. A sports specialist provided instruction on methodology and teaching strategies, ensuring the attendees understood how to guide each session, motivate the children, and make appropriate corrections. Sessions were also established to address common difficulties to ensure structured and effective implementation.

(6) Integration:

The sessions were presented in detail, outlining their structure and content based on the program, as well as the required circuits, times, and materials. Final modifications were incorporated, and the program was structured with standardized sessions twice a week. The adequacy and consistency of the new program with the original evidence-based program were preserved.

(7) Testing:

The training of the team to implement the program for the development of motor skills in five-year-old children involved the professionals reviewing the intervention manual and practicing the activities and intervention circuits in practical sessions with preschool children. This one-day in-person training was developed by the experts in the study. Finally, final adjustments were made before formal implementation.

(8) Monitoring:

The effectiveness of the functional program for the development of motor skills before and after the intervention in five-year-old children was evaluated. The effect on the motor skills of children who participated in the adapted functional program was compared with a control group (standard early childhood education curriculum).
