# Supplementary material for: Effect of an Intervention Program on Gross Motor Skills in Peruvian Preschool Children
Source: Children (Basel). 2026 Sep 8;13(9):1210. doi: 10.3390/children13091210 (PMC13605003; doi:10.3390/children13091210)
Supplement: Supplementary file 1 [file children-13-01210-s001.zip › children-4167417-supplementary.pdf]

Supplementary Material S1

Component	Description
**Specific types of activities performed**	Structured motor circuits combining locomotor skills (forward and lateral running, horizontal and vertical jumping, hopping, obstacle negotiation), object-control tasks (overhand and underhand throwing, catching with two hands, kicking stationary and moving balls, controlled dribbling), and stability exercises (single-leg balance, animal walks, controlled landings, core stabilization tasks). Activities were organized in sequential stations requiring coordination, agility, balance, and motor sequencing.
**Structure and duration of each session**	Each session lasted approximately 45 min and included: (a) 5 min of dynamic warm-up (light running, joint mobility, movement imitation), (b) 30 min of structured motor circuits organized into 4–6 stations, and (c) 5 min of cool-down including breathing exercises and guided stretching. Brief instructional transitions were incorporated between stations.
**Frequency and intensity**	The program was implemented over 8 weeks, with 2 sessions per week (16 sessions total). Intensity was moderate, characterized by continuous movement with short recovery periods between stations, adapted to the developmental level of five-year-old children.
**Progression across the intervention**	Activities were implemented following a consistent motor circuit structure across sessions. While the overall framework remained stable, natural variability in task execution and contextual adaptations (e.g., movement combinations and spatial organization) provided opportunities for progressive motor engagement throughout the intervention period.
**Materials and equipment used**	Cones for directional changes; agility rings and floor markers for foot-placement patterns; low hurdles (10–20 cm) for jumping tasks; soft foam balls (15–20 cm diameter) for throwing and catching; size-appropriate rubber balls for kicking and dribbling; coordination ladders; gym mats for floor and landing exercises; lightweight plastic sticks for target and control activities.
